# Advancing methods for comparative urban research: A city-centric protocol and longitudinal dataset for US metropolitan statistical areas

**DOI:** 10.1371/journal.pone.0316750

**Published:** 2025-03-31

**Authors:** Jason R. Jurjevich, Katie Meehan, Nicholas M. J. W. Chun, Greg Schrock

**Affiliations:** 1 School of Geography, Development & Environment, University of Arizona, Tucson, Arizona, United States of America; 2 Department of Geography, King’s College London, London, United Kingdom; 3 ECONorthwest, Portland, Oregon, United States of America; 4 Nohad A. Toulan School of Urban Studies and Planning, Portland State University, Portland, Oregon, United States of America; Tallinn University of Technology School of Engineering: Tallinna Tehnikaulikool Inseneriteaduskond, ESTONIA

## Abstract

Comparative urban research in the USA has an unacknowledged data and methodological problem at the metropolitan scale, rooted in geographic and definitional boundary changes of urban areas across time. In this article, we introduce a new spatial dataset, decision criteria, and methodological protocol for longitudinal and comparative research with US metropolitan statistical areas (MSAs)—known as ‘metros’—in a way that centers a ‘city-centric’ approach to comparison while significantly reducing spatial error and bias. First, we review gaps and limitations of existing approaches and identify three major but previously unacknowledged sources of error, including a new source of bias we call ‘spanning error.’ Next, we explain our methodological protocol and decision criteria, which are guided by the twin aims of reducing spatial bias and ensuring metropolitan consistency over time. We then introduce our improved dataset, which covers the 50 largest MSAs from 1980-2020. We argue that by centering the urban area as the fundamental unit of analysis—a city-centric approach—our methodology and dataset provides robust and dynamic metropolitan definitions that advance comparative urban studies while improving precision and accuracy in urban data analysis across different time scales. We discuss broader applications of our methodology and identify advantages and limitations over existing techniques, including potential applications of this work in policy, planning, and future research.

## Introduction

Comparative urban research in the USA has a data problem at the metropolitan scale. The problem is rooted in the simple fact that geographic and definitional boundaries of urban areas change over time. In this article, we introduce a new spatial dataset, decision criteria, and methodological protocol for longitudinal and comparative research with US metropolitan statistical areas (MSAs)—known as ‘metros’—in a way that centers a ‘city-centric’ approach to comparison while significantly reducing spatial error and bias.

**Fig 1:**
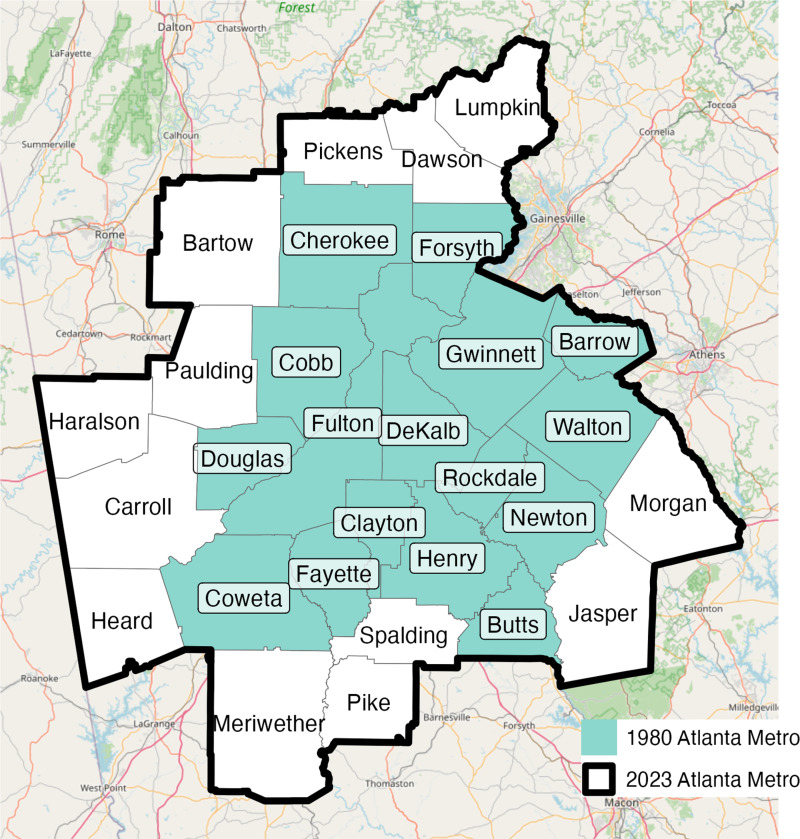
The urbanization of Atlanta, GA creates challenges for methods that use ‘static’ metro definitions. Source data: [3,4].

Population growth makes it difficult to develop consistent metropolitan definitions of Metropolitan Statistical Areas (MSAs). In Atlanta, for example, the population has more than tripled over the past 40 years, from just over 2 million residents in 1980 to 6.3 million in 2023 [1,2]. The official metropolitan footprint of Atlanta has grown to accommodate its urbanization, from 16 counties in 1980 to 29 counties in 2023 (Fig 1); yet any ‘static’ metro definitions do not keep pace with these normal urban transformations. This problem is easily overlooked or buried, and becomes problematic to draw comparisons across time and between cities. The Atlanta example powerfully illustrates how methods that do not account for urbanization—a core focus of urban studies—introduces spatial bias, error, and related problems that pose obstacles to high-quality research (Fig 1). The aim of this paper is to bring potential sources of spatial error to light, and to advance a new and improved dataset and methodology for comparative US metro research.

This article offers a new methodology and spatial dataset [5] that advances a ‘city-centric’ approach to longitudinal and comparative urban research in the USA. The article introduces a new methodological protocol, design principles, and spatial dataset that circumvents unacknowledged forms of spatial error, promotes a ‘dynamic’ (in contrast to ‘static’) method of urbanization, and better supports urban theory-building across time and space. We innovate a city-centric approach that (1) privileges the urban area (i.e., the MSA) over time, and that (2) accommodates the normal arc of growth, expansion, and change (i.e., urbanization) in the 50 largest US metro regions from 1980 to 2020. This methodology, we argue, offers distinct advantages in reducing error—including a new source of bias we call *spanning error*—and more accurately reflects the intellectual goals of many urban researchers, who seek to explain changing dynamics and trends over time in ways that preserve the larger social and economic functionality of the urban area (in this case, the MSA) as the principal unit of analysis.

To develop the methodology, our analysis uses census microdata: defined as data about households and individuals. Census microdata are one of the most powerful tools in the social science analytical toolbox. Unlike predefined census tables, microdata allow researchers to conduct customized analyses of a wide array of socioeconomic, health, and demographic phenomena affecting individuals, population groups, and communities [4,6]. Microdata are also useful for avoiding ecological inference issues, an all-too-common source of error that is introduced when researchers use aggregate data to make individual and household-level inferences [7]. In the United States, the building blocks of census microdata are public use microdata areas (PUMAs). Containing roughly 100,000 individuals, PUMAs are the smallest geography available for reporting census microdata, designed to ensure respondent data remain private.

Using census microdata, we published the first systematic and comprehensive analysis of household water access in US cities, highlighting the racial, class-based, and housing disparities of water insecurity [8]. We also released a companion dataset containing custom geographic definitions for the 50 largest US metros and accompanying code [9]. This article is an improvement on our 2020 scholarship as it (1) clearly identifies and quantifies examples of spatial bias in existing approaches; (2) updates the database—still available for public download and use—from 1980 to 2020; and (3) explicates a methodological protocol and set of decision criteria. In the next section, we review the methodological design of existing approaches and identify three significant but unacknowledged sources of spatial error, illustrated by examples. We then explain and justify the decision criteria used to develop our methodology, which is guided by the twin aims of reducing spatial bias (including spanning error) and ensuring metropolitan consistency over time—a city-centric approach. We then introduce our recoded open access dataset, which covers the 50 largest MSAs (1980-2020), and discuss key issues in recoding and database development. Finally, we discuss advantages and limitations of our approach in relation to existing techniques, and conclude with potential applications of this work in policy, planning, and research.

## Limitations of existing approaches

Definitional and boundary changes of metropolitan areas, combined with technical issues, are major impediments to precision and accuracy in longitudinal analysis. In this section, we review existing approaches and identify three major problems and sources of spatial error. Such issues, we explain, help underscore the importance of developing a methodology that puts the urban region at the heart of analysis, while ensuring MSA consistency over time.

The first and most common approach uses static metro definitions that are anchored to a fixed point in time, such as the use of a 2000 metro definition for a 2000-2020 longitudinal analysis (e.g., [10–12]). The second approach, which is more robust, creates dynamic metro definitions by accounting for changes to county and/or PUMA assignments (e.g., [13–15]). Below we outline the limitations of existing approaches and illustrate the accompanying gaps and limitations.

### Problem 1: Changing definitions of metro areas

In reviewing existing approaches, the first major problem is the changing definition of MSAs. With counties serving as the building blocks of MSAs, the US Office of Management and Budget (OMB) redefines MSAs after each decennial census, adding or removing counties based on population change and commuting patterns [16]. The OMB groups counties into core-based statistical areas (CBSAs) according to metro-centered definitions of urbanized areas: 50,000 or more people residing in a densely populated urban core. This means that the physical expansion of metropolitan areas, a normal process that occurs in nearly all major US urban areas, results in changing county composition every ten years. The US Census Bureau provides historical delineation files detailing the historical evolution of metro definitions since 1950 [17].

Researchers can account for these OMB changes by adopting a *static* or a *dynamic* definition of the metropolitan region. A static definition holds the geographic boundaries of the MSA constant across all time periods, usually by incorporating non-metropolitan counties that were added in subsequent years into earlier datasets. For example, the Bureau of Economic Analysis’ Local Area Personal Income and Employment data series going back to 1969 have all been re-coded based on the current metropolitan county composition [10]. In contrast, a dynamic metropolitan definition—such as our approach—follows urban theory to center the urbanized area as the fundamental unit of analysis, expanding the definition over time to account for the physical expansion.

A dynamic definition tends to overstate quantitative metrics such as employment and population growth of a metropolitan area, since the increase from one year to another may reflect the reclassification of previously rural residents or workers as urban. In contrast, a static definition would tend to overstate the relative influence of agricultural functions in the metropolitan area, and potentially mask rural-to-urban migration by counting non-metropolitan areas as metropolitan at times when they did not exhibit the economic characteristics of urban areas. Choosing a dynamic or static definition may depend on whether the researcher intends to compare nominal growth rates between places, or capture more compositional characteristics such as industrial or occupational composition.

A second and equally important implication of changing definition of MSAs is that the OMB has adopted different categorization schemes of metropolitan categories over the decades. In the 1990 Census, many MSAs that had previously been considered a single metropolitan area were disaggregated into a consolidated MSA (CMSA) and constituent primary MSAs (PMSA). For example, in the Chicago metropolitan region, three new suburban primary MSAs encompassing five counties were created for the 1990 Census (Aurora, Joliet, and Lake counties), only to be re-aggregated into the Chicago primary MSA for Census 2000. In the 2000s, the OMB introduced a new scheme entirely, called core-based statistical areas, which include metropolitan statistical areas as well metropolitan divisions, consolidated statistical areas, and micropolitan statistical areas.

### Problem 2: Changes in PUMA boundaries

The task of developing consistent MSA definitions would be considerably easier if the building blocks of the dataset were consistent over time. The PUMA, not the county, is the smallest geography for analyzing and reporting microdata, and therefore serves as the building block for metro-specific microdata analyses. PUMA boundaries are redrawn by state-level officials after each decennial census to account for population shifts and urban growth. Therefore, it is important for researchers using microdata to account for changes in PUMA boundaries, which is the second major obstacle to conducting longitudinal analysis of metropolitan areas.

Researchers at IPUMS (formerly Integrated Public Use Microdata Series), part of the Institute for Social Research and Data Innovation at the University of Minnesota, have attempted to resolve this problem by developing two variables that create a harmonized set of PUMAs over time. The first, CONSPUMA, provides consistent PUMA geographies for the 1980-2000 decennial censuses, while the second, CPUMA0010 (Consistent PUMA, 2000-2010), follows a slightly different method for creating consistent PUMA geographies for the 2000 and 2010 Censuses [18]. A map of current PUMA boundaries for the USA are available from IPUMS USA [19].

The problem here is two-fold: 1) the aim of the IPUMS approach is to achieve PUMA consistency, not MSA consistency, and 2) consistency is achieved by including as many as a dozen or more PUMAs into a single CONSPUMA (Consistent PUMA), resulting in a significant loss of spatial granularity in the analysis. We take inspiration from the IPUMS approach and apply it to a different geography—the MSA—to provide comparability over time.

### Problem 3: Spanning error

In the metro core, PUMAs generally nest within counties due to higher population densities. At the urban periphery, however, things are trickier. To achieve the minimum population threshold of around 100,000 individuals, PUMAs often include suburban and exurban counties within metro areas, along with adjacent non-metropolitan (rural) counties. In short, we identify a new form of bias called ‘spanning error’—a problem that emerges when PUMAs span across metropolitan and non-metropolitan counties, typically along the suburban fringe—as a third major obstacle for longitudinal research. Below we outline two interrelated issues, one geographic and the other related to data confidentiality, that emerge from PUMA spanning, making it impossible to develop consistent geographic definitions of metropolitan areas.

A major problem of spanning error is that each US metropolitan area presents a unique challenge and inconsistent level of bias. To illustrate the geographic dimensions of PUMA spanning and its difficulties, we present an example from the 2020 Census. Three cases of PUMA spanning are visualized and explained: 1) perfectly nested and best case scenarios (e.g., Buffalo); 2) moderately difficult cases (e.g., Baltimore); and, 3) messy cases (e.g., San Antonio).

**Fig 2:**
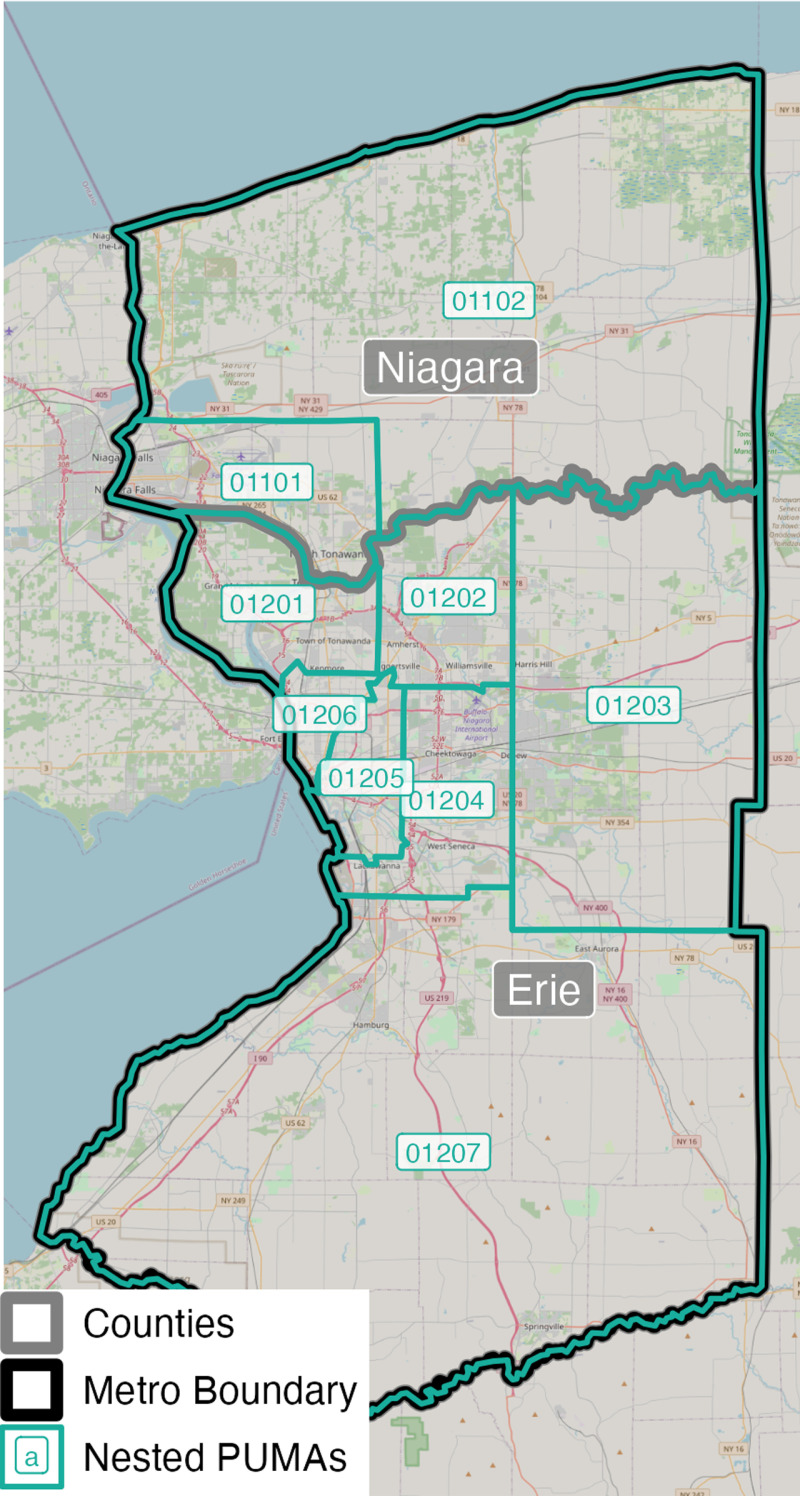
Example of Buffalo, NY, a perfectly nested and ‘best case’ scenario. Source data: [4].

The first example illustrates how PUMAs may nest perfectly within metro county boundaries, as in Buffalo, NY (Fig 2). The absence of PUMA spanning in Buffalo is a ‘best case’ scenario because the researcher does not have to subjectively assign PUMAs to metro geographies given that all PUMAs are wholly contained within the official metro boundary. In other words, the researcher can compare the Buffalo MSA across time without importing spatial bias.

**Fig 3:**
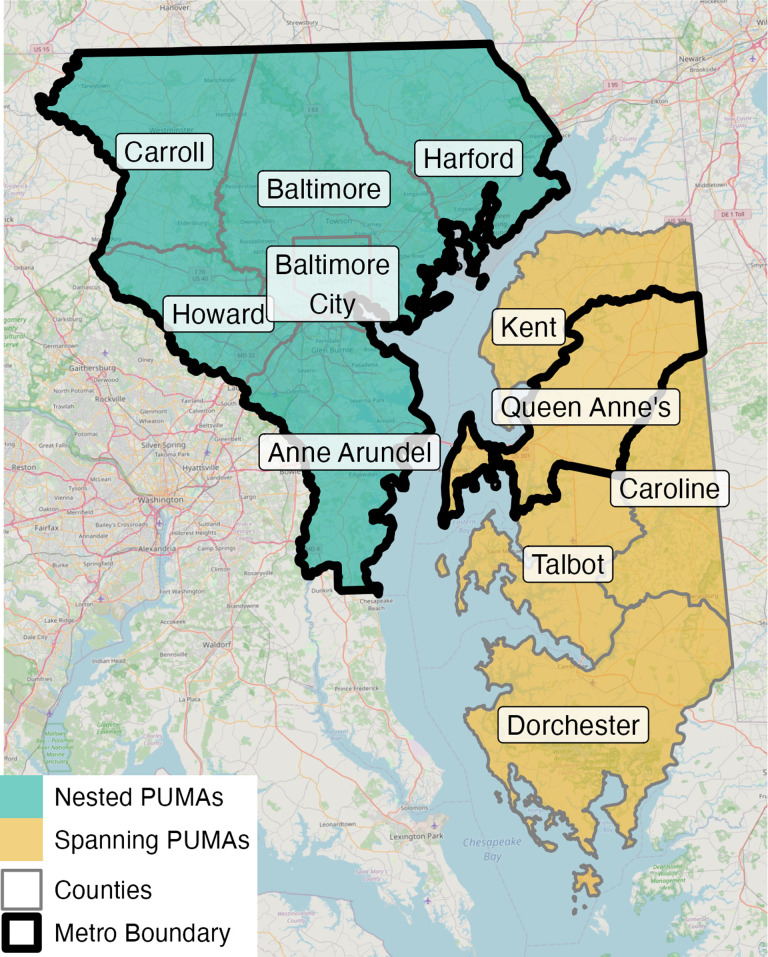
Example of Baltimore, MD, a ‘moderate case’ scenario. Source data: [4].

Unfortunately, the ‘best case’ scenario is uncommon. Researchers are more likely to encounter geographically discordant boundaries that require careful analysis and subjective researcher decisions. In moderately difficult cases such as Baltimore (Fig 3), Queen Anne’s County (part of the Baltimore MSA), located on Maryland’s Eastern Shore, is part of a PUMA that also includes four non-metro counties (Kent, Talbot, Caroline, and Dorchester). This geographic mismatch raises an important practical consideration: whether to include or exclude this PUMA in the Baltimore MSA. Fewer than three in ten individuals (29%) of the Eastern Shore PUMA lived in the Baltimore MSA in 2020, so excluding this PUMA makes sense given that most individuals reside in areas *outside* the Baltimore metro boundary.

**Fig 4:**
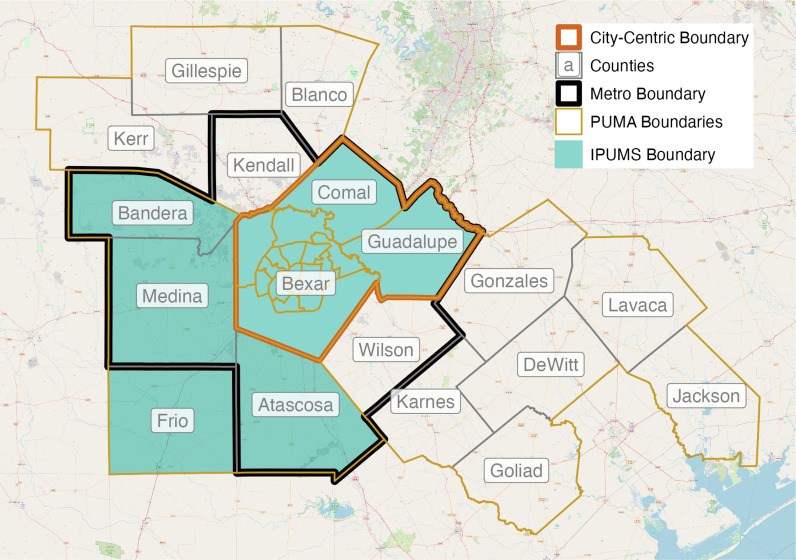
Example of San Antonio, TX, a ‘messy’ scenario. Source data: [4].

The third and final example, featuring San Antonio, TX, presents an extreme case of PUMA spanning (Fig 4). In San Antonio, there are *three* unique PUMA spans, producing a ‘messy’ situation that forces the researcher to make difficult decisions on PUMA assignments. The first span covers the western and southern part of the metro. Here, the metro counties of Atascosa, Medina, and Bandera are part of a PUMA that also includes one non-metro county (Frio). Most individuals (87%) in this PUMA reside within the San Antonio metro boundary. The second span, located in northwest San Antonio, includes Kendall County. The PUMA containing Kendall County also includes three non-metro counties (Kerr, Gillespie, and Blanco), with roughly one in three individuals (33%) residing in the San Antonio metro. The third span is located in southeastern San Antonio. This PUMA includes Wilson County and six non-metro counties (Karnes, Gonzales, Goliad, DeWitt, Lavaca, and Jackson), meaning that one in three (34%) individuals in this PUMA reside within the San Antonio metro.

Our analysis here underscores the importance of minimizing PUMA spanning, whenever possible. Currently, the Census State Data Center (SDC) coordinator from each US state redraws PUMA boundaries after each decennial census to account for population change [20]. By closely collaborating with metropolitan planning organizations and other stakeholders, this locally-driven process ensures that PUMAs are drawn in a way that supports regional transportation, economic, housing, and other planning efforts. The downside, however, is that PUMA boundaries often vary by state and over time, and span across metro and non-metro county boundaries, representing a significant obstacle for comparative urban research across the 50 metros.

A second major implication of PUMA spanning is that the Census Bureau codes all individual cases of ‘spanned’ PUMAs as “indeterminable” even though some individuals reside within a given metro area. Researchers analyzing housing affordability in Baltimore, for example, need to first select Baltimore metro residents from the census microdata. The problem is that residents from Queen Anne’s County—part of the Baltimore MSA—are *excluded* because they reside in a spanned PUMA coded as indeterminable. Recoding individual cases as “indeterminable” is designed to prevent researchers from determining the location of individual cases within a PUMA, thereby preserving respondent confidentiality, a federal mandate outlined in Section 9 of the Census Act, U.S. Code Title 13. Excluding these census records introduces a type of bias in urban research, which we refer to as *spanning error* (Table 1).

**Table 1:** 2020 Population spanning error, 50 largest US metros.

	PUMA 2020	Metro 2020	Spanning
	Population	Population	Error
Louisville, KY-IN	1,154,089	1,362,180	15.3%
Charlotte, NC-SC	2,301,953	2,660,329	13.5%
Richmond, VA	1,138,114	1,314,434	13.4%
Virginia Beach, VA-NC	1,544,391	1,780,059	13.2%
Oklahoma City, OK	1,246,225	1,425,695	12.6%
Raleigh-Durham, NC	1,769,559	2,002,893	11.6%
St. Louis, MO-IL	2,495,925	2,820,253	11.5%
Cincinnati, OH-KY-IN	2,020,642	2,249,797	10.2%
Denver-Boulder, CO	2,976,220	3,294,579	9.7%
Memphis, TN-MS-AR	1,218,018	1,345,425	9.5%
San Antonio, TX	2,343,531	2,558,143	8.4%
Austin, TX	2,140,272	2,283,371	6.3%
Washington, DC-VA-MD-WV	5,892,427	6,278,542	6.1%
Atlanta, GA	5,754,223	6,104,803	5.7%
Pittsburgh, PA	2,317,063	2,457,000	5.7%
Indianapolis, IN	1,987,059	2,089,673	4.9%
Minneapolis-St. Paul, MN-WI	3,515,256	3,690,261	4.7%
Columbus, OH	2,040,518	2,138,926	4.6%
Salt Lake City, UT	1,810,140	1,895,133	4.5%
Kansas City, MO-KS	2,105,894	2,192,035	3.9%
Nashville, TN	1,943,203	2,014,444	3.5%
San Jose, CA	1,936,259	2,000,468	3.2%
Portland, OR-WA	2,448,234	2,512,859	2.6%
Chicago, IL-IN-WI	9,249,650	9,449,351	2.1%
Jacksonville, FL	1,572,807	1,605,848	2.1%
Detroit, MI	4,303,422	4,392,041	2.0%
Boston, MA-NH	4,845,626	4,941,632	1.9%
Birmingham, AL	1,158,338	1,180,631	1.9%
Baltimore, MD	2,794,636	2,844,510	1.8%
Houston, TX	7,035,279	7,149,642	1.6%
Philadelphia, PA-NJ-DE-MD	6,180,214	6,245,051	1.0%
Hartford, CT	1,139,001	1,150,473	1.0%
Dallas-Fort Worth, TX	7,568,755	7,637,387	0.9%
Miami, FL	6,102,340	6,138,333	0.6%
New York, NY-NJ-PA	19,984,267	20,081,935	0.5%
Phoenix, AZ	4,835,022	4,845,832	0.2%
Buffalo, NY	1,166,902	1,166,902	0.0%
Cleveland, OH	2,185,825	2,185,825	0.0%
Las Vegas, NV	2,265,461	2,265,461	0.0%
Los Angeles, CA	13,200,998	13,200,998	0.0%
Milwaukee, WI	1,574,731	1,574,731	0.0%
New Orleans, LA	1,007,275	1,007,275	0.0%
Orlando, FL	2,673,376	2,673,376	0.0%
Providence, RI	1,676,579	1,676,579	0.0%
Riverside, CA	4,599,839	4,599,839	0.0%
Sacramento, CA	2,397,382	2,397,382	0.0%
San Diego, CA	3,298,634	3,298,634	0.0%
San Francisco, CA	4,749,008	4,749,008	0.0%
Seattle, WA	4,018,762	4,018,762	0.0%
Tampa, FL	3,175,275	3,175,275	0.0%

Source data: [4]. Note: the PUMA 2020 Population refers to the cumulative population of PUMAs that nest wholly within the metro boundary.

We calculated the 2020 population spanning error for the 50 largest US metros in Table 1. A first important takeaway is that spanning error is not trivial: in fact, spanning error is significant for many metropolitan areas. In Louisville, KY, almost 1 in 6 (15.3%) metro residents are not designated as living in the Louisville metro due to spanning error (Table 1). Roughly 1 in 3 metros have a 2020 population spanning error of at least 5%, and the error exceeds 10% in eight metros (Table 1). The magnitude of spanning error, combined with the geographic dimensions of the error (i.e., concentrated in the suburban and exurban peripheries of metro areas), has significant implications for descriptive and inferential analyses.

A second and equally important takeaway is that spanning error will vary depending on the variable analyzed. For example, while the *total population* spanning error in Dallas is 0.9% (Table 1), the spanning error is more than three times as high—roughly 3%— for the *non-Hispanic, White population*. Digging further into this issue to provide greater context, the spanning error in Dallas originates in one PUMA in the northwest part of the metro that spans outside of the official metro boundary. Given that individuals residing in this PUMA are classified as indeterminable, the microdata population estimate of non-Hispanic, White households is 3,699,000, which is 113,000 fewer than the official published census estimate (3,812,000). The difference, roughly 3% of the total metro area, represents spanning error. This case, which illustrates only one spanned PUMA, underestimates the potential severity of the statistical bias. If there are three or four spanned PUMAs, the statistical bias can sometimes approximate 10% or more of the official census estimate.

## Methods and analytical approach

### Reducing spanning error and ensuring consistency

We argue that centering the urban area as the fundamental unit of analysis—a ‘city-centric’ approach—is critical to advance empirical insights and theory-building in comparative urban research. To do so, we needed a methodology and dataset that provides robust and dynamic metropolitan definitions, which are essential for improving precision and accuracy. Our protocol established two goals. First, we aimed to *reduce bias* in metropolitan areas with ‘indeterminable’ data resulting from PUMA spanning across metro and non-metro areas. Second, we focused on *enhancing the overall consistency* across the time periods by creating dynamic and flexible metropolitan definitions that accommodated the urbanization process. Together, these goals allow us to develop a consistent set of metropolitan definitions that reflect urban expansion and definitional changes in boundaries over time.

These goals informed subsequent decision criteria that we used to determine whether to recode and include or exclude spanned PUMAs in the metropolitan dataset. We established four decision criteria to guide our methodology and database development, which we refer to as the ‘city-centric’ approach (Table 2).

**Table 2:** Metropolitan recoding criteria for PUMAs, City-Centric and IPUMS research approaches.

		Research Approaches	
	Criterion	City-Centric	IPUMS
1	Completeness	**X**	
2	Majority urban	**X**	**X**
3	90% cumulative population	**X**	
4	Consistency over time	**X**	

To achieve our first goal of *reducing bias from spanning error*, we calculated the total population of PUMAs that nest perfectly within metro boundaries. If the spanning error (i.e., missing population due to PUMA spanning) was less than 7% of the total metro population, we classified the metro dataset as ‘complete’ and no additional analysis was conducted for the given metro. If, however, the spanning error was greater than 7%, we classified the metro dataset as ‘incomplete’ and proceeded to the next criterion. Based on our analysis, 7% marked an inflection point in the data where the spanning error began increasing exponentially. For the second criterion, we prioritized PUMA recodes that restored the metro’s population. To avoid skewing the dataset in the opposite direction, we set a rough decision rule that the recoded PUMAs needed to contain at least 50% percent metropolitan observations (i.e., their inclusion would add more urban than rural population). To illustrate: in Baltimore, the Queen Anne’s County PUMA would not be prioritized as a recode under the second criterion because only 29% percent of the PUMA’s population resides in the Baltimore metropolitan area. The third criterion ensures that the cumulative population of the nested and recoded PUMAs represent at least 90% of the metro population (i.e., mirroring the 90% confidence interval used in statistical significance testing). Together, the three criteria provide cross sectional or point-in-time consistency for each metro across the study period, 1980-2020.

To *enhance definitional consistency*, we developed a fourth criterion, consistency over time. Unlike the others, this criterion is largely qualitative, allowing us to make nuanced and place-sensitive judgments about how and whether to recode PUMAs. To make place-sensitive judgments, we considered additional variables, maps, and other key information about a metro to support dynamic and flexible metropolitan definitions that prioritized longitudinal consistency. Visualizing geographic boundaries, for example, helped us first identify the geographic location of spanned PUMAs and second, prioritize recoding by reviewing census data to assess the similarity of demographics for counties of spanned PUMAs compared to the larger metropolitan area.

Together, these hybrid quantitative-qualitative criteria are foundational to our protocol, which we refer to as the ‘city-centric’ approach, and differs considerably from the IPUMS recoding philosophy and methodology. The city-centric approach prioritizes longitudinal consistency over all other criteria, which forced us to sometimes override logics established in the first three criteria if we determined excluding or including a PUMA would provide a more consistent metropolitan definition. We explain our decision making process below using three illustrative examples.

First, we added PUMAs to a metropolitan area that were mostly or entirely non-metropolitan if recoding created a more consistent county-based metropolitan definition over the study period. Second, we expanded three metro boundaries—Raleigh, Salt Lake, and Denver—to include their neighboring metropolitan areas, Durham, Ogden, and Boulder, respectively. These combined metros are more representative of broader economic connections, commuting patterns, and sociocultural ties that bind these regions, providing greater longitudinal consistency.

Finally, we also considered *aggregation of PUMAs for migration variables* as a third subjective criterion from 1990-2010. Since 1990, the Census Bureau has aggregated PUMAs for the migration or place of residence variable, which reports where an individual resided one or five years earlier (MIGPUMA variable). The rationale for creating MIGPUMAs, which typically comprise two or three ‘regular’ PUMAs, is because migration (as a highly selective process) requires more data protection to ensure the confidentiality of census respondents. This means that MIGPUMA spanning is more common than PUMA spanning across metro and non-metro counties, resulting in greater *bias* introduced by ‘incomplete’ cases. Therefore, we included non-metro spanned PUMAs in the metropolitan definition whenever they were combined with metropolitan PUMAs as part of the MIGPUMA variable. For example, in 2000, the Pittsburgh metro included PUMA 2001, which spanned metropolitan Beaver County and non-metropolitan Lawrence County. Because the Census Bureau aggregated PUMA 2001 and metropolitan PUMA 2002 (southern Beaver County) into Pennsylvania MIGPUMA 20, it is necessary to include Lawrence County into the Pittsburgh metropolitan definition to maintain metro consistency (see S1 Fig).

### The recoding process

Creating a new spatial dataset required processing two different types of recodes. The first were metropolitan area recodes, which involved reclassifying components of larger metropolitan areas that were later classified by OMB as part of a single metropolitan area. Fort Worth, TX is a great example. Originally classified as its own metropolitan area from 1990 to 2000, Fort Worth has since been reclassified as a “metropolitan division” within a unified Dallas-Fort Worth MSA. Therefore, we recoded Fort Worth as part of Dallas-Fort Worth metro for these two periods to ensure metropolitan consistency over time.

A second set of recodes were processed to *reduce bias* introduced by incomplete metropolitan areas resulting from PUMA spanning, following the decision criteria outlined in Table 2. We manually processed 176 separate metropolitan recodes (38 for 1980, 43 for 1990, 26 for 2000, 41 in 2010, and 28 in 2020). Recoding was straightforward in some cases, including our decision to recode the New Jersey PUMA equivalent encompassing Ocean, Hunterdon, Sussex and Warren Counties—all non-metropolitan in 1980—to the New York-Northern New Jersey metro for that year based on their subsequent incorporation into the metropolitan region for 1990. Others were more complex, requiring us to closely adhere to the recoding criteria.

**Fig 5:**
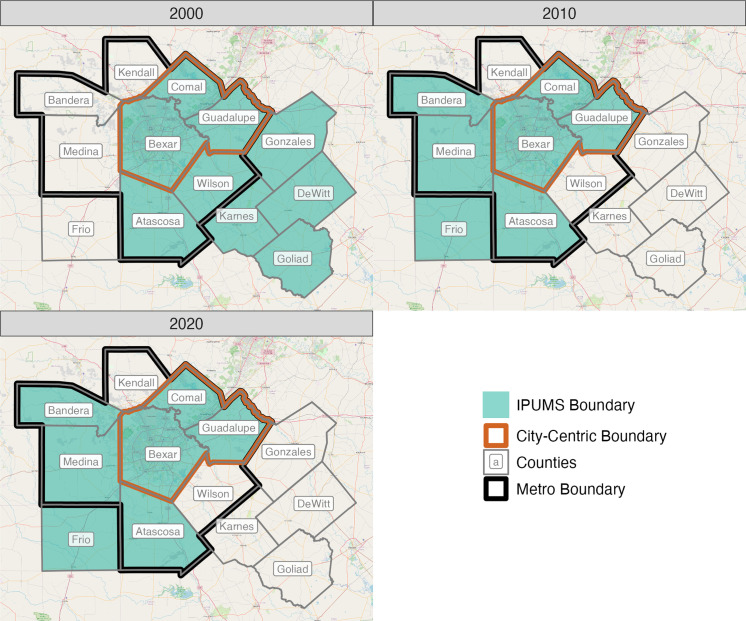
County-defined metro boundaries for San Antonio, TX, 2000-2020. Source data: Calculated by authors, [4].

To illustrate the challenging and often cumbersome nature of recoding process, we return to the example from San Antonio. In Fig 5, we present the official OMB county definition for the San Antonio metro (outlined in black), along with the corresponding City-Centric and IPUMS definitions for each decade during the 2000-2020 period. Between 1980 and 1990, Bexar (central core), Comal, and Guadalupe Counties comprised the San Antonio metro region; five counties (Atascosa, Bandera, Kendall, Medina, and Wilson) were added following the 2000 Census. Following our recoding criteria, we first determined that *three* unique PUMA spans in San Antonio resulted in a corresponding spanning error of 8.4% in 2020 (Table 1), rendering the metro incomplete (Criterion 1). However, given that the nested PUMAs in the three counties comprise at least 90% of the metro population (91.6%) (Criterion 3), we decided not to include additional spanned PUMAs in order to preserve longitudinal consistency (Criterion 4). Comparing our approach with IPUMS underscores the importance of this logic. By relying on one criterion—majority urban—to assign PUMAs to a given metro, the IPUMS protocol produces two different metropolitan definitions for San Antonio (i.e., the 2000 metro definition differs from the 2010-2020 definition) (Fig 5).

The decision logic and application of the four criteria across the 50 largest MSAs for the most recent census period, 2020, is presented in Table 3. Following our ‘city-centric’ approach, we processed PUMA recodes for 28 metropolitan areas in 2020. The accompanying rationale for including or excluding PUMAs is reported in Notes and Observations in Table 3. In best case scenarios like Buffalo where nested PUMAs account for at least 93% of the metro population, we considered the metro ‘complete’ and conducted limited additional analysis. There are some exceptions, however. For example, although Orlando is considered ‘complete,’ we added PUMA 11900 (Sumter County) to the Orlando MSA definition to ensure longitudinal consistency (Criterion 4). For ‘incomplete’ metros, we processed majority-urban (Criterion 2) PUMA recodes that restored at least 90% of the metro’s cumulative population (Criterion 3). To explain this process more thoroughly, we provide two illustrative examples. First, in Hartford, CT, we recoded PUMA 20500 (Lower Connecticut River Valley) to restore the cumulative population to 93% (Table 3). Second, returning to the San Antonio example, we decided to exclude the three spanned PUMAs (05500, 06000, and 06100) to preserve longitudinal consistency. Given that PUMAs 05000 and 06000 are majority-rural (34% and 33% urban, respectively) and do not meet Criterion 2, we only report the exclusion of the majority-urban PUMA (06100, 87% urban) in Table 3.

**Table 3:** Application of the City-Centric recoding criteria across the 50 largest US metros, 2020.

	Criterion 1: Completeness	Criterion 2: Majority Urban	Criterion 3: 90% Cumulative Population	Criterion 4: Consistency	Notes and Observations
Atlanta, GA	X				
Austin, TX	X			Yes	Consistency: added PUMA 05100 (Capital Area COG [East]-Bastrop, Caldwell, Fayette & Lee Counties)
Baltimore, MD	X				
Birmingham, AL	X			Yes	Consistency: added PUMA 01301 (Jefferson [Northwest] & Walker Counties)
Boston, MA-NH	X			Yes	Consistency: added PUMAs 00501 - 00507 [Worcester County], 01001 - 01004 [Bristol and Plymouth Counties], and excluded 00801 (Seacoast Region, Rockingham County [Southern] [NH])
Buffalo, NY	X				
Charlotte, NC-SC		^	97%	Yes	Completeness: added PUMAS 02700 (Lincoln and Cleveland [East] Counties), 00501 (Union and York [West] Counties [SC]), and 00700 (Chester, Fairfield, and Lancaster Counties [SC]); Consistency: added PUMAs 01900 (Davie, Yadkin, and Iredell [North] Counties and 03300 (Stanly and Cabarrus [East] Counties)
Chicago, IL-IN-WI	X				
Cincinnati, OH-KY-IN		^	99%		Completeness: added PUMA 03200 (Dearborn, Franklin, Ripley, Switzerland, and Ohio Counties [IN])
Cleveland, OH	X			Yes	Consistency: added PUMA 00900 (Ashtabula & Geauga Counties)
Columbus, OH	X				
Dallas-Fort Worth, TX	X				
Denver-Boulder, CO		^	99%	Yes	Completeness: added PUMAs 00401 (Foothills) and 00601 (Broomfield); Consistency: added PUMAs 01800 (Northeast Colorado), 01001 (Weld Rural), 01002 (Greeley), 001003 (South Weld), 00501 (East Boulder), 00502 (Boulder)
Detroit, MI	X				
Hartford, CT		^	93%		Completeness: added PUMA 20500 (Lower Connecticut River Valley)
Houston, TX	X				
Indianapolis, IN	X				
Jacksonville, FL	X			Yes	Consistency: added PUMA 10799 (Putnam & St. Johns [South] Counties)
Kansas City, MO-KS		^	100%+		Completeness; added PUMA 00800 (Johnson, Lafayette, Ray, Clinton & Caldwell Counties)
Las Vegas, NV	X				
Los Angeles, CA	X				
Louisville, KY-IN		^	100%+		Completeness; added PUMA 01800 (KIPDA Area Development District [Northeast])
Memphis, TN-MS-AR		^	91%	Yes	Nested PUMAs capture 91% of MSA population, but did not add additional PUMAs for consistency
Miami, FL	X			Yes	Consistency: added PUMA 08625 (Miami-Dade [South/Outside Urban Development Boundary] & Monroe Counties)
Milwaukee, WI	X				
Minneapolis-St. Paul, MN-WI		^	98%		Completeness: added PUMA 01500 (St. Croix & Dunn Counties [WI])
Nashville, TN		^	96%	Yes	Completeness: added PUMA 02300 (Dickson, Cheatham & Hickman Counties); Consistency: excluded PUMA 00600 (Macon, Dekalb & Cannon Counties)
New Orleans, LA	X				
New York, NY-NJ-PA	X			Yes	Consistency: added PUMAs 02803 (Dutchess County [North and East]), 02804 (Dutchess County [Southwest]), 02805 (Putnam County & Southern Dutchess County), 02901 (Orange County [Northeast]), 02902 (Orange County [Northwest], 02903 (Orange County [Southeast]), and excluded 00500 (Pike & Wayne Counties [NJ])
Oklahoma City, OK		^	94%	Yes	Completeness: added PUMA 21800 (South Central Oklahoma Counties); Consistency: added PUMA 21200 (East Central Oklahoma Counties)
Orlando, FL	X			Yes	Consistency: added PUMA 11900 (Sumter County)
Philadelphia, PA-NJ-DE-MD	X			Yes	Consistency: added PUMA 02501 (Salem & Cumberland [North] Counties [NJ])
Phoenix, AZ	X			Yes	Consistency: added PUMA 00400 (Gila, Graham, Greenlee Counties)
Pittsburgh, PA		^	96%		Completeness: added PUMA 04012 (Washington (South) & Greene Counties)
Portland, OR-WA	X			Yes	Consistency: excluded PUMA 07100 (Yamhill County)
Providence, RI			&	Yes	Consistency: excluded PUMAs 01001 (Bristol County-Attleboro, North Attleborough & Swansea [MA]), 01002 (Bristol County-Taunton, Easton & Mansfield [MA]), 01003 (Bristol County [Central]-Fall River, Somerset & Acushnet [MA}), and 01004 (Bristol County [South]-New Bedford, Dartmouth & Westport [MA])
Raleigh-Durham, NC		^	97%		Completeness: added PUMAs 01500 (Chatham & Lee Counties), 00400 (Granville, Person & Caswell Counties), and 00500 (Franklin & Vance Counties)
Richmond, VA		^	98%		Completeness: added PUMA 14900 (Crater Planning District Commission)
Riverside, CA	X				
Sacramento, CA	X				
Salt Lake City, UT	X			Yes	Consistency: added 03000 (Tooele & Box Elder Counties), 11002 (Davis County [South]), 11003 (Davis County [Northwest]), 11004 (Davis County [Northeast], 57001 (Weber County [West]), and 57002 (Weber County [East])
San Antonio, TX			92%	Yes	Consistency: excluded PUMA 06100 (Alamo Area COG [Southwest])
San Diego, CA	X				
San Francisco, CA	X				
San Jose, CA	X				
Seattle, WA	X				
St. Louis, MO-IL		^	98%		Completeness: added PUMAs 01700 (Franklin & Carter Counties), 11700 (Macoupin, Morgan, Jersey, Cass, Greene, Scott & Calhoun Counties [IL]), and 00400 (Lincoln, Warren, Audrain, Pike & Montgomery Counties)
Tampa, FL	X				
Virginia Beach, VA-NC	X				
Washington, DC-VA-MD-WV		^	98%		Completeness: added PUMAs 06100 (Rappahannock-Rapidan Regional Commission [VA]), 17700 (George Washington Regional Commission [South] [VA]), and 01501 (Calvert & Southeast St. Mary’s Counties [MD])

Notes: 1) &-Nested PUMAs capture roughly 91% of the MSA population, but our definition includes 65% of the MSA population because PUMAs 01001-01003 are included in the Boston, MA MSA for consistency. 2) A map of current PUMA boundaries for the USA are available from IPUMS USA [19].

### Evaluating the city-centric and IPUMS approaches

**Fig 6:**
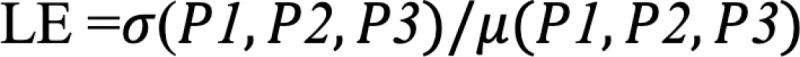
Longitudinal error (LE) equation.

We evaluated the accuracy of the city-centric and IPUMS approaches in supporting our twin goals—reducing spatial bias and simultaneously ensuring definitional consistency over time—by calculating the longitudinal error (Table 4). Here, longitudinal error measures the consistency of PUMA-defined metropolitan boundaries using the 2020 Census population. We calculated the longitudinal error by first estimating the standard deviation of the 2020 metro population statistics for each decade across the 2000-2020 period (i.e., *P*1, , *P*2, *P*3). Next, we express the longitudinal error as a percentage by dividing the standard deviation by the mean 2020 metro population statistic. The longitudinal error (LE) equation is expressed mathematically as (Fig 6):

**Table 4:** Longitudinal Error, City-Centric and IPUMS approaches.

	Longitudinal Error		
	City-Centric	IPUMS	Difference
**Greater Consistency Using City-Centric**			
Oklahoma City, OK	0.9%	5.2%	*4.3%*
Kansas City, MO-KS	1.2%	5.4%	*4.2%*
New Orleans, LA	9.8%	13.7%	*3.9%*
Cleveland, OH	0.0%	2.7%	*2.7%*
Providence, RI	0.2%	2.3%	*2.1%*
Portland, OR-WA	0.0%	2.0%	*2.0%*
Denver-Boulder, CO	0.2%	2.2%	*2.0%*
St. Louis, MO-IL	1.0%	2.7%	*1.7%*
Atlanta, GA	0.1%	1.4%	*1.3%*
Boston, MA-NH	0.5%	1.7%	*1.2%*
New York, NY-NJ-PA	1.0%	1.7%	*0.7%*
San Antonio, TX	0.0%	0.5%	*0.5%*
Nashville, TN	3.6%	3.9%	*0.3%*
Phoenix, AZ	0.5%	0.8%	*0.3%*
Tampa, FL	0.5%	0.8%	*0.3%*
San Diego, CA	0.4%	0.6%	*0.2%*
Virginia Beach, VA-NC	0.0%	0.1%	*0.1%*
Miami, FL	1.0%	1.1%	*0.1%*
**Mean (Median)**	**1.2% (0.5%)**	**2.7% (1.9%)**	
**Greater Consistency Using IPUMS**			
Charlotte, NC-SC	8.3%	3.9%	*4.4%*
Jacksonville, FL	3.6%	0.2%	*3.4%*
Birmingham, AL	5.0%	2.5%	*2.5%*
Raleigh-Durham, NC	5.2%	2.9%	*2.3%*
Orlando, FL	2.8%	1.1%	*1.7%*
Philadelphia, PA-NJ-DE-MD	1.3%	0.3%	*1.0%*
Washington, DC-VA-MD-WV	1.2%	0.4%	*0.8%*
Chicago, IL-IN-WI	1.5%	1.1%	*0.4%*
Pittsburgh, PA	2.8%	2.5%	*0.3%*
**Mean (Median)**	**3.5% (2.8%)**	**1.7% (1.1%)**	
**No Difference**			
Austin, TX	0.6%	0.6%	*0.0%*
Baltimore, MD	0.4%	0.4%	*0.0%*
Buffalo, NY	0.2%	0.2%	*0.0%*
Cincinnati, OH-KY-IN	3.1%	3.1%	*0.0%*
Columbus, OH	0.0%	0.0%	*0.0%*
Dallas-Fort Worth, TX	0.3%	0.3%	*0.0%*
Detroit, MI	1.6%	1.6%	*0.0%*
Hartford, CT	3.6%	3.6%	*0.0%*
Houston, TX	0.4%	0.4%	*0.0%*
Indianapolis, IN	1.4%	1.4%	*0.0%*
Las Vegas, NV	0.0%	0.0%	*0.0%*
Los Angeles, CA	0.1%	0.1%	*0.0%*
Louisville, KY-IN	0.2%	0.2%	*0.0%*
Memphis, TN-MS-AR	1.2%	1.2%	*0.0%*
Milwaukee, WI	0.0%	0.0%	*0.0%*
Minneapolis-St. Paul, MN-WI	3.5%	3.5%	*0.0%*
Richmond, VA	2.3%	2.3%	*0.0%*
Riverside, CA	0.0%	0.0%	*0.0%*
Sacramento, CA	0.1%	0.1%	*0.0%*
Salt Lake City, UT	4.0%	4.0%	*0.0%*
San Francisco, CA	0.2%	0.2%	*0.0%*
San Jose, CA	0.0%	0.0%	*0.0%*
Seattle, WA	0.9%	0.9%	*0.0%*

Source data: Calculated by authors, [4].

**Fig 7:**
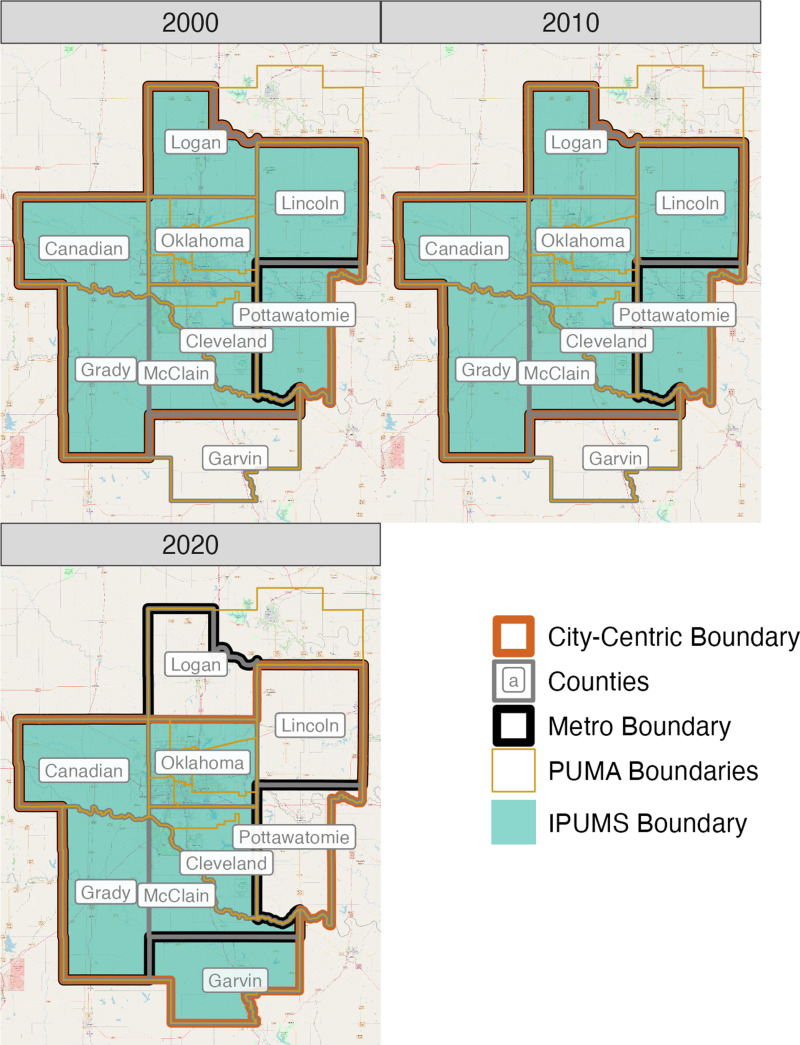
City-Centric and IPUMS metro boundaries for Oklahoma City, OK, 2000-2020. Source data: Calculated by authors, [4].

In Oklahoma City, for example, the metro boundaries are consistent between 2000 and 2010 under the city-centric and IPUMS approaches; there is a difference, however, in the 2020 definitions (i.e., the city-centric definition includes the PUMA containing Lincoln and Pottawatomie Counties) (Fig 7). As a result, the city-centric definitions returned 2020 metro population statistics of 1,498,149, 1,498,149, and 1,474,250 while the IPUMS definitions yielded in 1,498,149, 1,498,149, and 1,368,338 in 2000, 2010, 2020, respectively. Comparing these statistics to the official 2020 population of 1,425,695 (S1 Table), the standard deviation of the metro population error across these three periods under the city-centric approach (13,798), divided by the mean statistic (1,490,183), returns a longitudinal error of 0.9%. The IPUMS approach returned a longitudinal error of 5.2% for Oklahoma City (Table 4).

The results from Table 4 confirm that the city-centric approach provides more robust and dynamic metropolitan definitions that improve precision and accuracy in urban data analysis across time. The city-centric approach produced greater longitudinal consistency and less error in 18 metros, including Oklahoma City. The IPUMS approach, on the other hand, yielded more longitudinally consistent geographies and less error in 9 metros, likely due to the prioritization of MIGPUMAs in the city-centric approach. Also, the relative magnitude of the longitudinal error was lower under the city-centric approach (i.e., the mean longitudinal city-centric error was 2.1 times higher [3.5%/1.7%] compared to 2.3 times higher for IPUMS [2.7%/1.2%]). There was no difference in longitudinal error between the two approaches for roughly half of metropolitan areas (23 of 50).

## Discussion and conclusion

Comparative urban research is essential for understanding social and spatial change in a rapidly urbanizing world. Key to advancing this scholarship is a commitment to developing techniques and approaches in the methodological toolbox that support multi-sited comparisons, are sensitive to place and spatial difference, and support theory-building. Within the USA, as we have demonstrated, the failure to address geographic and definitional boundary changes reproduces spatial error and bias—including spanning error—that severely affects results. This problem is an issue of data justice given that spatial error and bias produces greater relative error for smaller-scale conditions (e.g., plumbing incompleteness, migration) and subpopulations (e.g., racial/ethnic populations, same-sex households), making it more difficult for researchers to draw inferences for populations facing discrimination and marginalization [21,22].

Until this point, researchers at IPUMS are the only scholars who have attempted to wrestle with these issues, developing several potential workarounds. Taking inspiration from the IPUMS approach, we developed a methodological protocol and decision criteria that offers two key advantages for scholars conducting comparative research in the largest metropolitan areas across the USA. First, the ‘city-centric’ methodology yields greater analytical accuracy, as illustrated in Table 4, by mitigating spatial bias and spanning error. Second, by centering the urban area as the fundamental unit of analysis—not the PUMA—the city-centric framework is a better pathway for providing consistent, yet dynamic metropolitan definitions over time that prioritizes longitudinal consistency.

Moving forward, our analysis demonstrates the importance of minimizing PUMA spanning in US Census policy and practice. The most obvious solution to reducing spanning error and simultaneously improving greater longitudinal consistency is to draw PUMAs in a way that prioritizes nesting PUMAs within 1) county boundaries, and 2) MSA boundaries. The good news is that both of these criteria were outlined by the US Census Bureau in a 2020 PUMA delineation document [23]. However, there are four issues that make it difficult for drawing longitudinally consistent PUMAs: 1) PUMAs may include noncontiguous areas, 2) some places, like New England, use town, not county boundaries for drawing PUMAs, 3) insufficient coordination with tribal, local, and state governments, community advocacy groups, policymakers and researchers, as well as regional planning agencies, resulting in PUMA boundaries that fail to account for various use cases, and 4) the need for the US Census Bureau to broaden and diversify PUMA delineation outreach. This can take two forms. First, PUMA delineation documents should, for example, contain simple and more direct language, illustrative examples for *each* PUMA criterion, and outline criteria that supports drawing “good” PUMA boundaries. Second, more careful attention needs to be paid to the repercussions of PUMA boundaries for applied and scholarly research. Addressing these issues are essential for realizing better-drawn PUMA boundaries, resulting in less error and making for more robust, data-driven policy.

Urbanization is a core feature of contemporary life in the USA and worldwide. Toward that end, this article innovates a new methodological approach and dataset that privileges the spatial patterns and processes of urbanization in ways that rigorously account for error, reflect the goals of many urban researchers, and support analytical approaches that pay attention to populations facing institutionalized discrimination and marginalization—a key goal of inclusive urbanization in SDG 11 (Sustainable Cities and Communities). While our approach is tailored to the data and context of the USA, future research might explore similar boundary issues and methodological problems in other countries experiencing rapid urbanization.

Future research should explore boundary issues and methodological problems in other countries experiencing urbanization. Approaches in comparative urban geography and sociology, for example, have generated important scientific insights to support problem-solving in matters of public health, housing, sustainability policy, migration, and education [11,24–28]. With almost 60% of the world’s population living in cities in 2022 [29], our work helps advance techniques and methodological tools that support theory-building about our changing urban environments.

## Supporting information

S1 Table 50 largest U.S. metros by population, 2020. (DOCX)

S1 Fig County-defined boundaries and selected PUMAs (and MIGPUMA) for Pittsburgh, PA, 2000. (TIF)
