# Supplementary material for: Advancing methods for comparative urban research: A city-centric protocol and longitudinal dataset for US metropolitan statistical areas
Source: PLoS One. 2025 Mar 31;20(3):e0316750. doi: 10.1371/journal.pone.0316750 (PMC11957320; doi:10.1371/journal.pone.0316750)
Supplement: S1 Table — (DOCX) [file pone.0316750.s001.docx]

**Supporting information**

|  | **Metro Area** | **2020 Population** |
| --- | --- | --- |
| 1 | New York, NY-NJ-PA | 20,081,935 |
| 2 | Los Angeles, CA | 13,200,998 |
| 3 | Chicago, IL-IN-WI | 9,449,351 |
| 4 | Dallas-Fort Worth, TX | 7,637,387 |
| 5 | Houston, TX | 7,149,642 |
| 6 | Washington, DC-VA-MD-WV | 6,278,542 |
| 7 | Philadelphia, PA-NJ-DE-MD | 6,245,051 |
| 8 | Miami, FL | 6,138,333 |
| 9 | Atlanta, GA | 6,104,803 |
| 10 | Boston, MA-NH | 4,941,632 |
| 11 | Phoenix, AZ | 4,845,832 |
| 12 | San Francisco, CA | 4,749,008 |
| 13 | Riverside, CA | 4,599,839 |
| 14 | Detroit, MI | 4,392,041 |
| 15 | Seattle, WA | 4,018,762 |
| 16 | Minneapolis-St. Paul, MN-WI | 3,690,261 |
| 17 | San Diego, CA | 3,298,634 |
| 18 | Denver-Boulder, CO | 3,294,579 |
| 19 | Tampa, FL | 3,175,275 |
| 20 | Baltimore, MD | 2,844,510 |
| 21 | St. Louis, MO-IL | 2,820,253 |
| 22 | Orlando, FL | 2,673,376 |
| 23 | Charlotte, NC-SC | 2,660,329 |
| 24 | San Antonio, TX | 2,558,143 |
| 25 | Portland, OR-WA | 2,512,859 |
| 26 | Pittsburgh, PA | 2,457,000 |
| 27 | Sacramento, CA | 2,397,382 |
| 28 | Austin, TX | 2,283,371 |
| 29 | Las Vegas, NV | 2,265,461 |
| 30 | Cincinnati, OH-KY-IN | 2,249,797 |
| 31 | Kansas City, MO-KS | 2,192,035 |
| 32 | Cleveland, OH | 2,185,825 |
| 33 | Columbus, OH | 2,138,926 |
| 34 | Indianapolis, IN | 2,089,673 |
| 35 | Nashville, TN | 2,014,444 |
| 36 | Raleigh-Durham, NC | 2,002,893 |
| 37 | San Jose, CA | 2,000,468 |
| 38 | Salt Lake City, UT | 1,895,133 |
| 39 | Virginia Beach, VA-NC | 1,780,059 |
| 40 | Providence, RI | 1,676,579 |
| 41 | Jacksonville, FL | 1,605,848 |
| 42 | Milwaukee, WI | 1,574,731 |
| 43 | Oklahoma City, OK | 1,425,695 |
| 44 | Louisville, KY-IN | 1,362,180 |
| 45 | Memphis, TN-MS-AR | 1,345,425 |
| 46 | Richmond, VA | 1,314,434 |
| 47 | Birmingham, AL | 1,180,631 |
| 48 | Buffalo, NY | 1,166,902 |
| 49 | Hartford, CT | 1,150,473 |
| 50 | New Orleans, LA | 1,007,275 |

**S1 Table.** **50 largest U.S. metros by population, 2020**. Source data: [1].

**S1 Figure. County-defined boundaries and selected PUMAs (and MIGPUMA) for Pittsburgh**, **PA, 2000**. Source data: Calculated by authors, [4].

References

1. US Census Bureau [Internet]. Suitland (MD): 2020 Decennial Census, DEC Redistricting Data (PL 94-171), Table P1 (Race). 2020 [cited 2024 Oct 9]. Available from: https://data.census.gov/table/DECENNIALPL2020.P1?q=P1: TOTAL POPULATION
