# Supplementary figures and images for: Advancing methods for comparative urban research: A city-centric protocol and longitudinal dataset for US metropolitan statistical areas
Source: PLoS One. 2025 Mar 31;20(3):e0316750. doi: 10.1371/journal.pone.0316750 (PMC11957320; doi:10.1371/journal.pone.0316750)

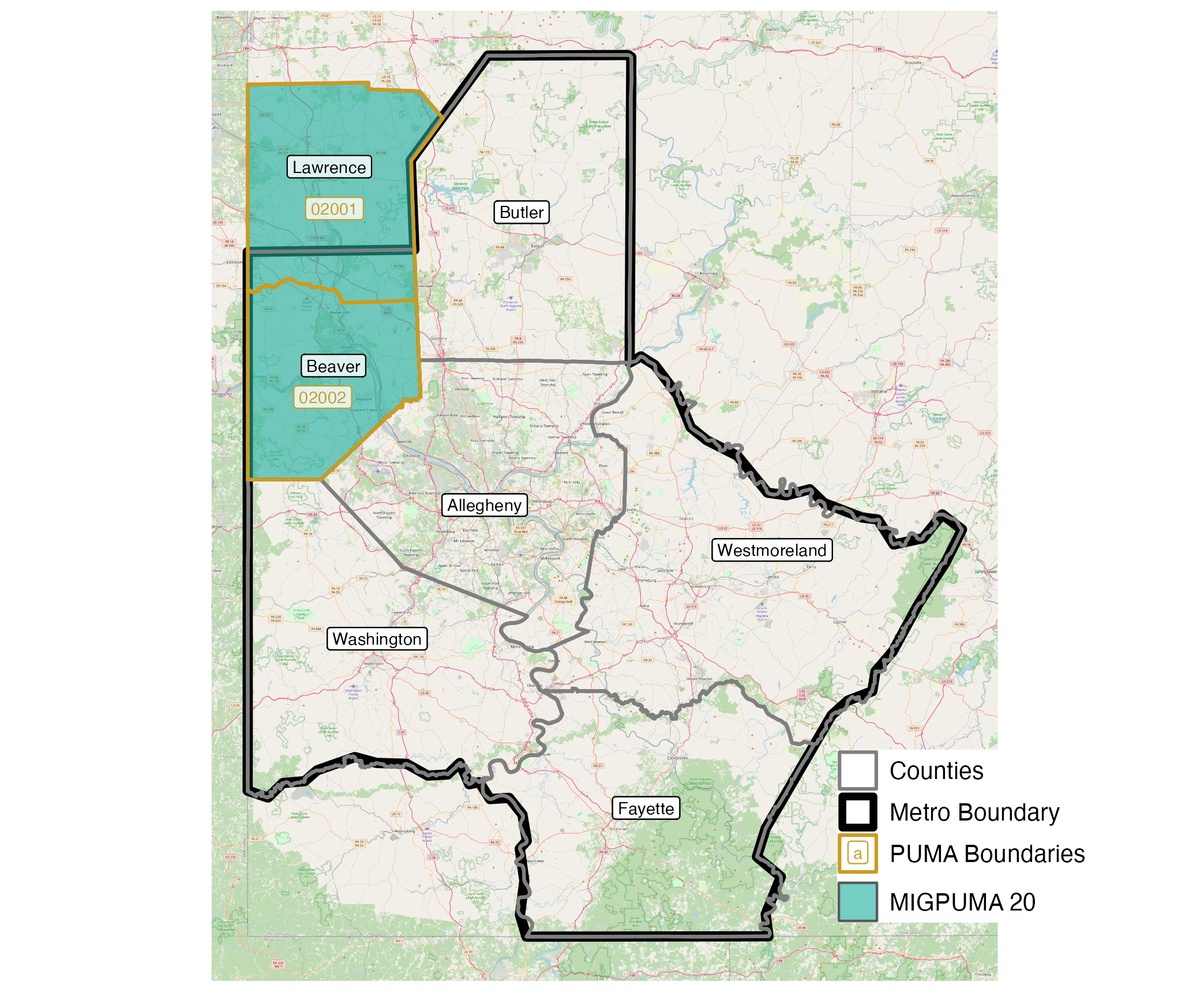

Supplement: S1 Fig — (TIF) [file pone.0316750.s002.tif]
